# Studies on Dual Helmholtz Resonators and Asymmetric Waveguides for Ventilated Soundproofing

**DOI:** 10.3390/s24051432

**Published:** 2024-02-22

**Authors:** Inkyuk Han, Inho Lee, Gwanho Yoon

**Affiliations:** Department of Manufacturing Systems and Design Engineering, Seoul National University of Science and Technology, Seoul 01811, Republic of Korea; 18102067@seoultech.ac.kr (I.H.); inholee@seoultech.ac.kr (I.L.)

**Keywords:** noise control, ventilation, dual Helmholtz resonators, asymmetric waveguides, aperiodic resonance, acoustic metamaterial

## Abstract

Achieving the simultaneity of ventilation and soundproofing is a significant challenge in applied acoustics. Ventilated soundproofing relies on the interplay between local resonance and nonlocal coupling of acoustic waves within a sub-wavelength structure. However, previously studied structures possess limited types of fundamental resonators and lack modifications from the basic arrangement. These constraints often force the specified position of each attenuation peak and low absorption performance. Here, we suggest the in-duct-type sound barrier with dual Helmholtz resonators, which are positioned around the symmetry-breaking waveguides. The numerical simulations for curated dimensions and scattered fields show the aperiodic migrations and effective amplifications of the two absorptive domains. Collaborating with the subsequent reflective domains, the designed structure holds two effective attenuation bands under the first Fabry–Pérot resonance frequency. This study would serve as a valuable example for understanding the local and non-local behaviors of sub-wavelength resonating structures. Additionally, it could be applied in selective noise absorption and reflection more flexibly.

## 1. Introduction

Acoustic noise is an undesirable result of all living and industrial activities. Parallel to the increasing number of population and diversity of devices, the property of interested noise constantly changes [1,2]. In contrast, the conventional noise reduction method during the transmission stage remains static: blocking the path or space near the noise sources with sound absorbing materials (SAMs) [3,4]. SAMs generally adhere to the mass-density law, resulting in linear absorption performance for the low-frequency range [5]. Lower interactions between long-wavelength waves and structure lead to less energy dissipations; thicker and heavier SAMs are required for sufficient noise reduction. Thus, achieving simultaneity in addressing low-frequency noise and ventilation requirements poses a significant challenge.

Acoustic metamaterials (AMMs) are artificial structures composed of purposely arranged resonators in a sub-wavelength scale. With the resolution criterion by wave property, AMMs are equated with the effective medium of the same volume that owns effective mass density *ρ* and effective bulk modulus *κ* [6,7]. Unlike previous chemically categorized ordinary materials, the strong dispersion of resonating components results in a complex range of effective properties: positive, negative, and near-zero values [8,9,10,11,12]. These unprecedented properties open up new possibilities for promising research topics such as acoustic focusing [13,14,15], acoustic clocking [16,17,18], acoustic communications [19,20,21], and acoustic absorbers [22,23].

Over the past decade, ventilated soundproofing with AMMs has been conceptualized and developed as duct noise approaches [24,25,26,27]. For an in-duct structure, the transverse bilayer concept has been widely implemented, featuring a central passage and surrounding space-coiled half-wavelength resonators [28,29,30]. Continuum state waves from the former and discrete state waves from the locally resonating latter result in destructive interferences, known as Fano-like interference, occurring at periodic frequency points. Reflective domains are broadened between the points by the non-local coupling of waves [31,32]. While this structural type offers great advantages in terms of simple design and broadband transmission loss, its reduction is periodically restrained and dependent on reflection.

Another common approach involves a symmetric central passage surrounded by end-closed locally resonating structures such as space-coiled quarter-wavelength resonators (QWRs) [33,34,35] and Helmholtz resonators (HRs) [36,37]. Under the first Fabry–Pérot resonance point, the mutual response of the contracted central passage and each resonator yields partial reflective and absorptive domains [38]. However, much of previous research tended to suggest attenuation performance without a clear classification of them. Only the less of them discussed the effective control of the absorptive domain [35]. Therefore, achieving noise control with efficient absorption performance requires ongoing trials and discussions on various fundamental local resonators and arrangement-induced non-local interactions.

Here, we conceptually present an in-duct type hollow acoustic structure with dual Helmholtz resonators (DHRs) and asymmetric waveguides for wave manipulation. These properties lead to over 90% attenuation of incident sound energy across the two frequency ranges; one is from 550 Hz to 874 Hz and the other is from 1344 Hz to 2500 Hz, owning the absorptive domains from 542 Hz to 707 Hz and 1344 Hz to 1688 Hz. Notably, these two absorption bands are influenced by the accumulated and reinforced two aperiodic resonance peaks of each DHR within asymmetric arrangements. Thus, this study may be instructive in finding the relation between the fundamental resonator and its arrangement in sub-wavelength resonating structures. Additionally, it would mark the beginning of utilizing absorptive domains for reducing non-regular peak noise in various application scenarios.

## 2. Design and Simulation for Acoustic Metastructure

**Figure 1:**
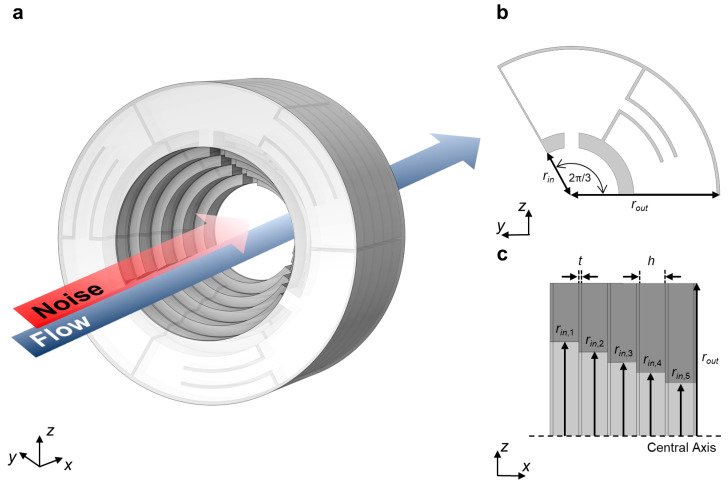
Schematics of designed metastructure with certain parameters. (**a**) Conceptual view of the designed metastructure. (**b**) Radial plane (*yz* plane) view of the layer, focusing on the unit DHR. Outer radius *r_out_* = 50 mm and inner radius *r_in_*, which varies through the layer. (**c**) Axial plane (*zx* plane) view of the designed metastructure. The thickness of the general wall *t* = 1 mm and the height of unit DHR *h* = 8.8 mm.

Noise and flow undergo soundproofing and ventilation as they traverse the designed structure in Figure 1a. The ventilation of the background medium stems from the central orifice. Effective attenuation occurs through the acoustical response of the orifice and the surrounding resonators. The orifice behaves as a Fabry–Pérot resonator, which transmits all the energy when it periodically resonates. Between the passing points, it reflects the most incident energy due to the mismatched impedance. This transmissive and reflective property results in an insufficient absorption coefficient. The effective absorption response would derive from the surrounding resonators. It induces additional resonance under the first Fabry–Pérot resonance, where an asymmetric reflection takes place. This profile, called Fano-like interference, escorts the effective absorption coefficient at the dip of reflection response. The magnitude of the absorption coefficient would vary depending on the design of the resonator in each case [35,36].

To manage the absorption performance, we introduce the DHR as a fundamental resonator. One single Helmholtz resonator (SHR) is connected to the central passage with radius *r_in_*, while the other is radially connected to the cavity of the previous one. This configuration molds the DHR of the outer radius *r_out_*, as depicted in Figure 1b. The DHR defines its two resonance frequencies based on its dimension parameters [39]. It leads to a great degree of freedom in setting the resonance frequencies, compared to the restrained peak position of the QWR only by its length. When the DHR responds to these frequencies, massive dissipation would take place in each neck region. This characteristic indicates that DHR enables the thorough control of absorption peaks by precisely selected dimensions.

The designed structure comprises a stack of five layers, whose inner radii are narrow over the +*x* direction, as depicted in Figure 1c. Each layer accommodates the three DHRs in a radial sequence. With these configurations, we promote an increase in the absorption coefficient due to two aspects. One is the asymmetric shape that defines the scattering coefficient dissimilarly based on the incident propagate direction. This definition divides the reflection coefficient by the incident direction, which contributes to controlling the absorption coefficient in a more unconstrained way. The other is critical damping in a DHR or a specific layer. This concept happens when the following layer functions as an effective rigid wall, which reflects the incident energy. This backward energy would be efficiently dissipated if the front layer could resonate with the reflected wave [40].

The designed resonator and metastructure are numerically simulated by using the pressure acoustic module in COMSOL Multiphysics^TM^ 6.1. To estimate the noise attenuation performance, we conduct the frequency domain study. Scattering coefficients are gained through the plane wave ports, which are located at both ends of the cylindrical waveguides. A large impedance difference between air and structural material leads every wall to be acoustically rigid. We account for the intrinsic losses from viscous and thermal dissipation, which mainly occurs at neck sections. Those domains are assigned to the narrow-region acoustic. With this simulation set-up, only the material property of air was employed as follows: density *ρ_air_* = 1.29 kg/m^3^, speed of sound *c_air_* = 331 m/s, Prandtl number Pr = 0.71, ratio of specific heats *γ* = 1.4, dynamic viscosity *η* = 1.839 × 10^−5^ kg m^−1^ s^−1^ and atmosphere pressure P_atm_ = 101,325 Pa.

The numerical simulation in Section 3.1 focuses on studying the side-branched unit DHR with open region radius *r_in_* = 16 mm. In Section 3.2, two types of models were simulated. One aims to investigate the band diagram of the designed structure. Only the air domain, which fills the inside of the structure, was modeled. Periodic boundary condition was selected on the inlet and outlet, which simplifies the interested wave vector into only the *x* direction. The other type of model is for calculating scattering responses for in-duct structure. It includes the cylindrical waveguide, which is extruding in both directions from structures along the *x* direction (see details in the Supplementary Materials).

## 3. Results and Discussion

### 3.1. Dual Helmholtz Resonators

**Figure 2:**
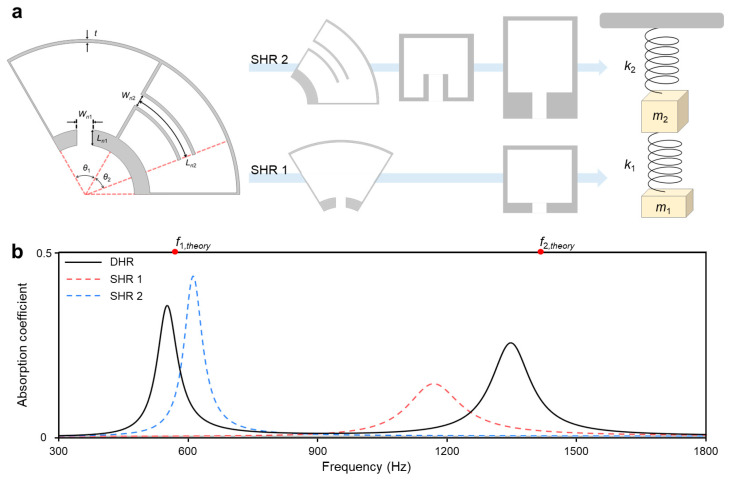
Design and absorption coefficient of unit DHR. (**a**) Approximation of designed unit DHR into connected two pairs of a mass-spring system. One pair is from SHR 1 with the following parameters: central angle of cavity *θ*_1_ = 60°, neck width *W_n_*_1_ = 5 mm, and neck length *L_n_*_1_ = 5 mm. The other is from SHR 2 with the following parameters: central angle of the cavity 120°—*θ*_1_, neck width *W_n_*_2_ = 4 mm, central angle of the embedded neck *θ*_2_ = 40°, and corresponding neck length *L_n_*_2_ = ((*r_in_* + *L_n_*_1_ + *r_out_* − *t*)/2) × *θ*_2_ × (π/180) = 24.43 mm. (**b**) Absorption coefficient of unit DHR, SHR 1, and SHR 2. Two red dots represent the analytical resonance frequency of DHR.

Large-scale differences in wavelength and structure dimension allow for the simplification of wave analysis as a harmonic oscillator [41]. Especially for the resonating structure, it approximates the forced damped system with equivalent excitation strength, mass element, and spring element [42]. These properties lead the DHR to the mass-spring–mass-spring model, as illustrated in Figure 2a. This equivalency results in the finding that the resonance frequency of DHR corresponds to the natural frequency of the oscillating model. The connected mass in each neck of *i*th SHR *m_i_* would satisfy Equations (1) and (2) by Newton’s second law and the following variables: angular frequency *ω*, the density of air *ρ_air_*, speed of sound *c_air_*, oscillating displacement of neck mass in each SHR *x*_i_, the cross-sectional area of the neck in *i*th SHR *A_ni_*, and effective resonating volume of *i*th SHR *V_i_* [39].

(1) $$-m_{1}\omega^{2}x_{1}+\frac{c_{air}^{2}\rho_{air}A_{n1}^{2}}{V_{1}}x_{1}-\frac{c_{air}^{2}\rho_{air}{A_{n1}A}_{n2}}{V_{1}}x_{2}=0$$

(2) $$-m_{2}\omega^{2}x_{2}+\frac{c_{air}^{2}\rho_{air}^{2}A_{n2}^{2}}{V_{1}}x_{2}+\frac{c_{air}^{2}\rho_{air}^{2}A_{n2}^{2}}{V_{2}}x_{2}-\frac{c_{air}^{2}\rho_{air}^{2}{A_{n1}A}_{n2}}{V_{1}}x_{1}=0$$

The effective length of the neck in *i*th SHR *l’_ni_* was introduced, which replaces the notation mass as $m_{i}=\rho_{air}\;A_{ni}\;{l\prime }_{ni}$ (see details in the Supplementary Materials). This replacement binds Equations (1) and (2), which results in the matrix form. It represents Hooke’s law with the sequence of **kx** = **F**. The eigenvalue of the given stiffness matrix would correspond to the resonance frequency of the DHR, as shown in Equation (4).

(3) $$\left[\begin{array}{cc} -\omega^{2}+\frac{c_{air}^{2}A_{n1}}{{l\prime }_{n1}V_{1}} & -\frac{c_{air}^{2}A_{n2}}{{l\prime }_{n1}V_{1}}\\ -\frac{c_{air}^{2}A_{n1}}{{l\prime }_{n2}V_{1}} & -\omega^{2}+\frac{c_{air}^{2}A_{n2}}{{l\prime }_{n2}V_{1}}+\frac{c_{air}^{2}A_{n2}}{{l\prime }_{n2}V_{2}} \end{array}\right]\left[\begin{array}{c} x_{1}\\ x_{2} \end{array}\right]=\left[\begin{array}{c} 0\\ 0 \end{array}\right]$$

(4) $$f_{1,2}=\frac{c_{air}}{2\sqrt{2}\pi }\sqrt{\left(\frac{A_{n1}}{{l\prime }_{n1}V_{1}}+\frac{A_{n2}}{{l\prime }_{n2}V_{1}}+\frac{A_{n2}}{{l\prime }_{n2}V_{2}}\right)\pm \sqrt{{\left(\frac{A_{n1}}{{l\prime }_{n1}V_{1}}+\frac{A_{n2}}{{l\prime }_{n2}V_{1}}+\frac{A_{n2}}{{l\prime }_{n2}V_{2}}\right)}^{2}-4\frac{A_{n1}}{{l\prime }_{n1}V_{1}}\frac{A_{n2}}{{l\prime }_{n2}V_{2}}}}$$

The dissipation, which comes from massive energy exchange to the external, would occur at the resonance state. It implies that the absorption peaks are located near the analytically defined resonance frequency in Equation (4). Numerical simulations were conducted for each SHR and unit DHR with assigned dimension values for the validation. All resonators were connected in a side-branched way to solely measure the absorption coefficient. Figure 2b represents its result, which adheres to the theory of point-symmetric scatter. This theory imposes a limitation on the maximum value of the absorption coefficient to 1/2 [43]. In the comparison of analytical resonance frequency (red dot) and numerical absorption peak frequency (black line), they are located close together with some deviation. The common deviation might stem from the value of the end-corrected length. A relatively large deviation on *f*_2_ comes from the unique resonance of SHR 1. As the shorter neck length of SHR 1, it has a larger resonance frequency than SHR 2. When those SHRs connect and SHR 1 resonates, not only the neck and cavity of SHR 1, but the part of SHR 2 neck also resonates as an effective volume. It results in the approximation of resonating volume, which manifests the deviation.

**Figure 3:**
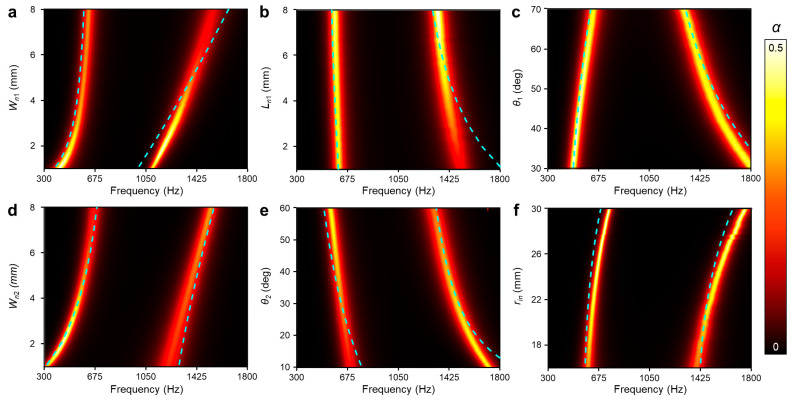
The absorption coefficient (*α*) by the varying geometric parameters. The dashed line for the analytical resonance frequency. (**a**,**b**) Parameters related to the neck of SHR 1: width *W_n_*_1_ and length *L_n_*_1_, respectively. (**c**) Circular sector angle of the first cavity *θ*_1_. (**d**,**e**) Parameters related to the neck of SHR 2: width *Wn*_2_ and length factor *θ*_2_, respectively. (**f**) Open region radius *r_in_*.

The transition and amplification of absorption peaks are confirmed through the geometrical studies shown in Figure 3. The cyan dashed line in Figure 3 represents the resonance frequency of unit DHR through the parametric study for each dimension. All trends align closely with the absorption peaks of the numerical calculations. It implies that the designed unit DHR maintains the property of the connected two Helmholtz resonators even with the geometric changes. This consistent analysis underscores the notion that the selection of parameters determines the response of the lossy mechanical system in each scenario. Parameters are categorized based on their impact on the structure following this discussion.

Dimensions, which are related to each neck part, induce not only frequency shifts but also absorption amplification. A narrower neck width shifts both peaks to a lower frequency by following Equation (4). The second peak of the absorption coefficient shows a causal tendency with changes in *W_n_*_1_, leading to larger losses by resonating in the smaller space. This relation is also valid between *W_n_*_2_ and the first peak of the absorption coefficient. Longer neck length exhibits a comparable effect to the narrower neck width. The dimensions of the cavity would mainly induce the shifts in peak frequency on the same principle. *θ*_1_ and *r_in_* are the variables that only change the volume of each cavity. They mainly exhibit the variation in resonance frequency, not in the amplitude of the absorption coefficient. Change in *θ*_1_ leads to the volume fraction between two cavities in limited space: *θ*_1_ and *θ*_2_ = 120° − *θ*_1_. This results in non-parallel shifting of resonance points, which could be either converged or dispersed. Alternations in *r_in_* bring parallel shifting, allowing for the changes in each cavity volume in the same way. These findings suggest that DHR could offer flexibility in dimension selection when a certain property of absorption coefficient is required.

### 3.2. Resonators in Asymmetric Waveguides

Metastructures often use multilayered fundamental resonators to enhance both the magnitude and bandwidth of the target property [27,38]. These improvements stem from the non-local properties, which originate from the interactions among closely located resonators. We employ the concept of symmetry-breaking structures to enhance the absorption performance. The asymmetry configuration defines the scattering coefficient differently depending on the direction of the incident wave. This opens up the possibility of attaining zero values in two eigenvalues of the scattering matrix, which theoretically corresponds to perfect absorption [40]. An elevated absorption coefficient could be achieved with the resonators that simultaneously suppress both transmission and reflection. These suppressions are realized through the effective reflecting wall and critical coupling, respectively. Within the asymmetry configuration, the rear-located resonator acts as a reflecting wall just above its resonance frequency. The front-located resonator, which matches its impedance to the surroundings in that frequency, shows the critical coupling [44].

**Figure 4:**
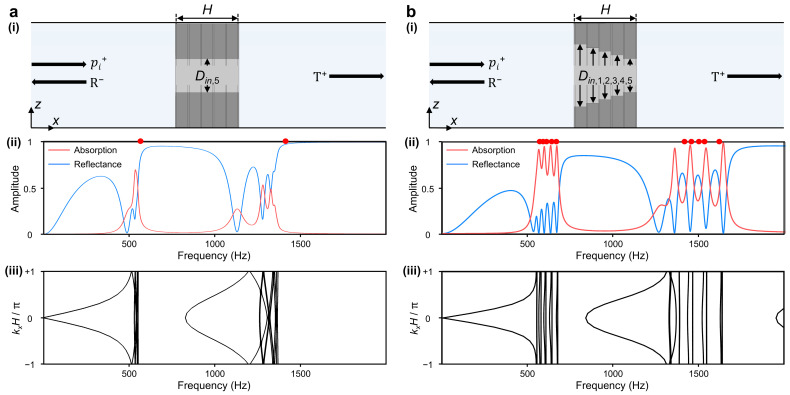
The soundproofing performance of two different waveguide structures. (**a**) Symmetric central passage with constant inlet diameter. (**b**) Asymmetric central passage with decreasing inlet diameter along the +*x* direction. In each section, (i) depicts the layer arrangement and wave propagation along the *x* direction, and (ii) presents the absorption coefficient (red line) and reflectance (blue line). The red dots display the analytical resonance frequency of DHRs, which are mounted in layers. Additionally, (iii) plots the corresponding band diagram of unit structure with total height *H* (*H* = 5*h* + 6*t*).

To implement the absorption amplification in asymmetrical structures, we conducted a comparative analysis between the two models. Each model consists of five layers, where three DHRs are accommodated in every layer. It generates two absorptive peaks in low and high frequencies. One model has a constant *D_in_*, while the other has varying *D_in_*, as depicted in Figure 4. These two models share the same dimensions for DHRs, except only for the value of *D_in_*. The inlet diameter of each layer *D_in,i_* is assigned as Table 1. Gradient modification in *D_in,i_* ensures not only the structural asymmetry, but also suggests the evenly spaced resonance frequency *f*_1,2_ by analytical predictions. The *D_in_* increment toward *D_in_*_,1_ layer gets smaller to ensure the properly spaced resonance. This diminishing trend reflects the varying change rate in cavity volume, which is relative to the initial dimension of it. Even a small deviation in larger *D_in_* results in a relatively large increment in the cavity volume.

The structure with constant *D_in_* exhibits insufficient and collapsed absorption peaks, which also deviated from the predicted resonance frequency of DHR, as in Figure 4a. This deficiency arises from the inadequate accumulation of strong dispersion. It necessitates infinitely accumulated layers along the +*x* direction for quasi-perfect absorption performance [40], which might come down to the tradeoff between thickness and absorption coefficient. The structure in Figure 4b achieved evenly spaced absorption peaks, which align well with the planned resonance and overcome the previous tradeoff. This specificity could be elucidated with the suggested band spectrum. The flat band represents the resonance state corresponding to each absorption peak. The bandgap suppresses the transmission of the wave while reflecting it. In Figure 4b, the structure shows the alternations of flats band and band gap in the band spectrum. This dispersion relation proves that asymmetrically arranged layers encompass the effect of reflecting wall and critical coupling effects.

**Figure 5:**
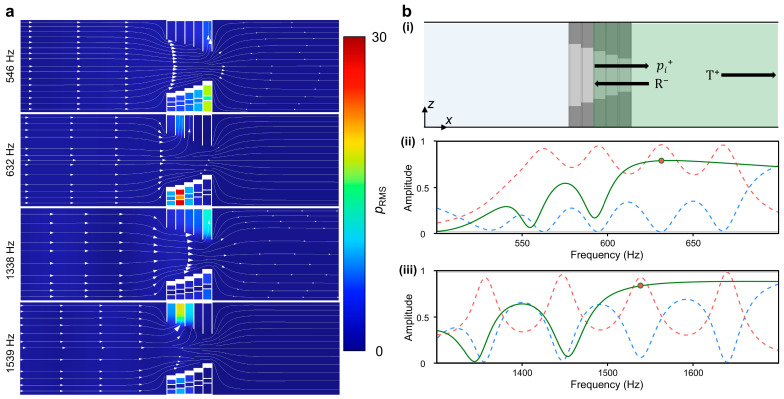
Acoustical response of the simulated structure. (**a**) Axial plane view of the sound field at certain frequencies. Intensity streamlines and *p*_RMS_ distribution fields are depicted. (**b**) (i) Modifications in the analysis domain (green region), where the incident wave starts at the boundary of *D_in_*_,2_ and *D_in_*_,3_ layers. Calculated reflectance (green line) is suggested with the (ii) first and (iii) second absorptive domain of full-waveguide simulation. The red and blue dashed lines correspond to the absorption coefficient and reflectance of full-waveguide simulation, respectively. The red dot indicates the reflectance of modified simulation at the specific frequency, where the third absorption peak was observed in the full-waveguide simulation.

**Figure 6:**
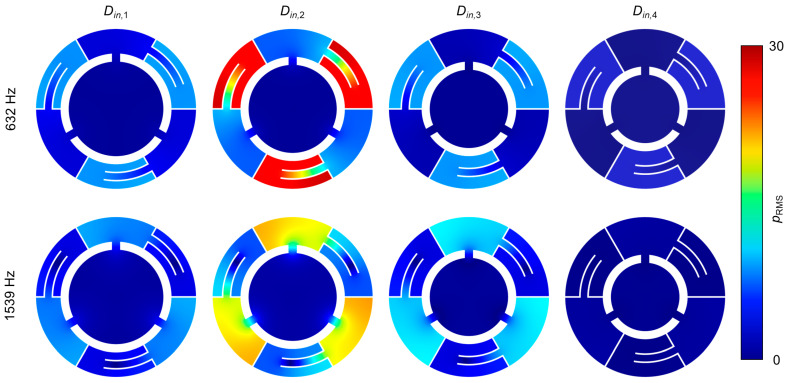
Radial plane view of each layer. The color map stands for *p*_RMS_ distribution under the frequency of 632 Hz and 1539 Hz.

Numerical simulation for the band spectrum requires the assumption of the periodic arrangement. This analytic restriction would bring a lack of reliability in practical explanations; further studies were conducted on acoustical response in the general waveguides. Figure 5a displays the two-dimensional cut plane containing the axial line. The color map illustrates the root mean square (RMS) value of acoustic pressure, and the streamline depicts the flow of acoustic intensity. Observing the flow of acoustic intensity, a significant reduction in scale is apparent across the structure at frequencies of 632 Hz and 1539 Hz. This corresponds to the third peak of the absorption coefficient at low and high frequencies, suggesting that a significant amount of energy might be dissipated through the structures. The *D_in_*_,2_ layer shows the outstanding responses at this frequency, and more detailed local property will be discussed in Figure 6. In the frequencies of 546 Hz and 1539 Hz, intensity flow passes through the structure and maintains its scale. The feeble resonance was observed in the *D_in_*_,5_ layer, which has the lowest resonance frequency and absorption performance.

The dissipation of intensity can be explained with the principal concepts of the designed structure: effective reflecting and critical coupling. Adjustments in analyzing domains were made by port selections, as seen in Figure 4b. This domain selection aims for delicate comprehension of non-local interactions between the layers. Strong reflection was observed at the point where the third absorption peak would be placed, as depicted in Figure 5b. These results prove that the amount of energy is reflected from the rear layer to the front layer, which is the concept of an effective reflecting wall. The activating layers in Figure 5a contain the local property of this structure with the concept of critical coupling. In the frequencies of 546 Hz and 1338 Hz, the layer of *D_in_*_,5_ has relatively minor excitations. This response is insufficient for critical coupling but adequate for acting as an effective reflecting wall. The first absorption peak would be placed just above this frequency, where the layer of *D_in_*_,4_ critically resonates. With these sequences, the four absorption peaks in low and high frequencies are generated.

Figure 6 depicts the mid-plane of each layer at the previously studied frequencies: 632 Hz and 1539 Hz. The color map illustrates the RMS value of acoustic pressure, so the part of SHR 2 responds to the lower resonance, and the part of SHR 1 responds to the higher resonance. As these frequencies are above the resonance frequency of the *D_in_*_,3_ layer, feeble resonances are presented as light blue color. From the strong reflection, which occurs between the *D_in_*_,2_ and *D_in_*_,3_ layer, the *D_in_*_,4_ layer with deep blue does not display any reactions. In the opposite direction, intensive resonances are presented in the *D_in_*_,2_ layer, which yields the peak of the absorption coefficient. These localized representations explain the principle of critical coupling and match well with Figure 5a.

### 3.3. Performance and Functionality

The designed metastructure in Figure 4b exhibits a low absorption coefficient at the local minimum points between the peaks. More closely spaced peaks would compensate for these low values but would result in a narrower bandwidth. We address this efficient-bandwidth tradeoff by varying the unit DHR in each layer. The three DHRs in each layer are labeled as DHR*_ij_*, which represents DHR in *i*th layers and *j*th order in radial sequences. This subscription is also valid for its related dimensions.

The width of SHR 2 neck *W_n_*_2_ was selected as the geometrical parameter and assigned, as shown in Table 2. The dimension remained constant at 4 mm for the fifth layer (*i* = 5) and first order (*j* = 1) of the remaining layer (*i* = 1, 2, 3, and 4). This consistency helps replicate the absorption peaks observed in the previous structure, thereby potentially allowing for additional absorption peaks with the rest selections. The other values were curated considering the distance between adjacent resonance frequencies (see details in the Supplementary Materials).

**Figure 7:**
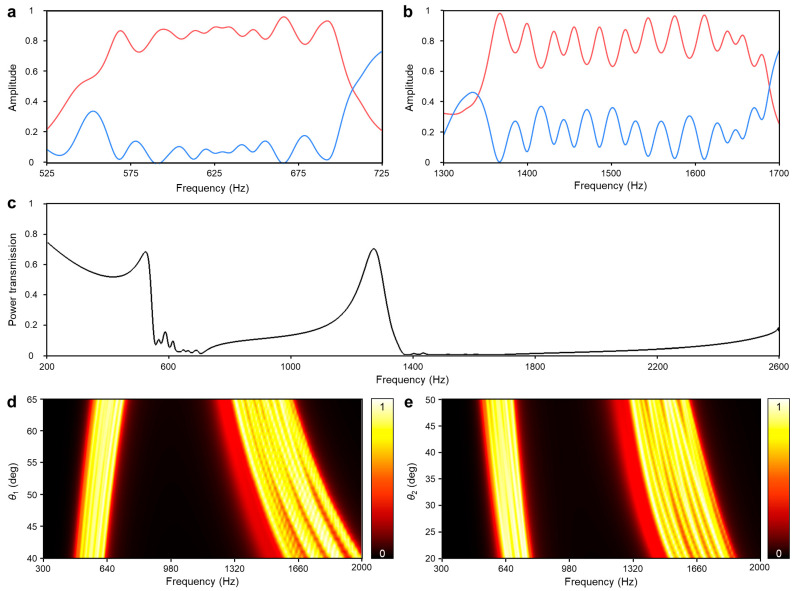
The noise insulation performance of the acoustic metastructure with individually adjusted DHRs. (**a**) First and (**b**) second absorptive domains with absorption coefficient (red line) and reflectance (blue line). (**c**) Transmittance of the optimized structure. (**d**) The absorption coefficient (*α*) by the varying geometric parameters: circular sector angle of the first cavity *θ*_1_. (**e**) The absorption coefficient (*α*) by the varying geometric parameters: length factor of second neck *θ*_2_.

The narrow spacing between each low-frequency absorption peak results in an ambiguous but gentle absorptive band from 550 Hz to 707 Hz, as seen in Figure 7a. The evenly spaced twelve high-frequency absorption peaks create the second absorptive band from 1344 Hz to 1688 Hz in Figure 7b. After these two absorptive regions, the reflective region follows behind the Fano-like interferences. Their collaboration results in over 90% attenuation of the incident sound energy across 550 Hz to 874 Hz and the other from 1344 Hz to 2500 Hz, as shown in Figure 7c.

Apart from the already adjusted geometric parameters, the remaining dimensions would be fine-tuned to enhance further functionality. The entire structure exhibits a similar shifting tendency to the unit DHR when the geometric parameters are varied. The altering value of *θ*_1_ shifts the two absorptive regions in a non-parallel way, while the altering value of *θ*_2_ shifts the two absorptive regions in a parallel way, as presented in Figure 7d,e. These two distinct shifting methods could serve as valuable tools for effectively mitigating irregular or widely spreading peak noises in real-world applications.

## 4. Conclusions

In summary, we suggest the ventilated soundproofing structure wherein unit DHRs are arranged in symmetry-breaking configurations. The DHR exhibits great flexibility in shifting two resonance frequencies and enhancing their absorption coefficients through numerous dimension variables. At the sub-wavelength scale, each DHR layer is asymmetrically loaded in the waveguides, functioning as both an effective reflecting wall and an effective dissipator based on the frequency of the incident wave. This leads to a significant amplification of absorption performance with four peaks in the low- and high-frequency domains. By adjusting the remaining dimensions in a gradient sequence, the metastructure demonstrates two attenuation bands, comprising absorptive and reflective regions. These absorption bands, originating from unit DHRs, leverage its advantages, such as shape versatility and unparalleled frequency shifting. Thus, this study may pave the way for utilizing the controllable absorptive domains for peak noise reduction in diverse application scenarios, along with the reflective domains.

## Figures and Tables

**Table 1:** *D_in_* of each layer (unit: mm) and corresponding resonance frequency (unit: Hz).

Layer Num	1	2	3	4	5
*D_in,i_* (=2 × *r_in,i_*)	58	54	48	42	32
*f*_1_	665	638	608	588	568
*f*_2_	1618	1562	1499	1455	1415

**Table 2:** Geometrical parameters of *W_n_*_2,*ij*_ (unit: mm).

*W_n_*_2,*ij*_	*i* = 1	2	3	4	5
*j* = 1	4	4	4	4	4
2	4.3	4.7	4.6	4.6	4
3	4.7	5.5	5.2	5.2	4

## Data Availability

Data are contained within the article.

## Supplementary Materials

The following supporting information can be downloaded at: https://www.mdpi.com/article/10.3390/s24051432/s1, S.1: Detailed description for numerical simulation; S.2: Detailed description for numerical simulation; S.3: Resonance frequency of the DHRs.
