# Supplementary material for: Studies on Dual Helmholtz Resonators and Asymmetric Waveguides for Ventilated Soundproofing
Source: Sensors (Basel). 2024 Feb 22;24(5):1432. doi: 10.3390/s24051432 (PMC10934021; doi:10.3390/s24051432)
Supplement: Supplementary file 1 [file sensors-24-01432-s001.zip › sensors-2823629-supplementary.pdf]

sensors

MDPI

Supplementary Materials for

Studies on Dual Helmholtz Resonators and Asymmetric Waveguides for Ventilated Soundproofing

Inkyuk Han, Inho Lee and Gwanho Yoon *

Department of Manufacturing Systems and Design Engineering, Seoul National University of Science and Technology, Seoul 01811, Republic of Korea; 18102067@seoultech.ac.kr (I.H.); inholee@seoultech.ac.kr (I.L.)
* Correspondence: gwanho@seoultech.ac.kr

S.1 Detailed Description for Numerical Simulation

Table S1. Details on numerical models.

Num	3D Model	Target	Simulation Options
1	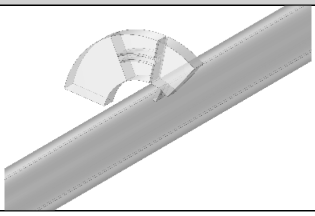	• Scattering field<br>• Section 2.1<br>• Figure 2<br>• Figure 3	• Side-branched DHR<br>• Port boundary condition for inlet and outlet
2	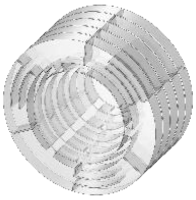	• Band spectrum<br>• Section 2.2<br>• Figure 4 (iii)	• Inlet-varying<br>• Same DHR in each layer<br>• Periodic boundary condition for inlet (src) and outlet (dst)<br>• Parametric study for k_x
3	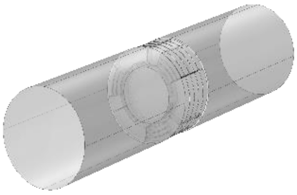	• Scattering field<br>• Section 2.2<br>• Figure 4 (ii)<br>• Figure 5<br>• Figure 6	• Inlet-varying<br>• Same DHRs in each layer<br>• Port boundary condition for inlet and outlet
4	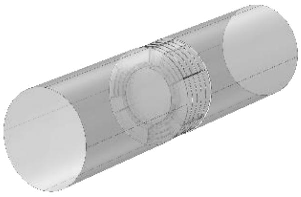	• Scattering field<br>• Figure 7	• Inlet-varying<br>• Gradient dimension for W_{n2} for each DHR in each layer<br>• Port boundary condition for inlet and outlet

S.2 Effective Parameters for Analytical Resonance Frequency

For the calculation of the resonance frequency of a dual Helmholtz resonator, some two parameters are used in Equation (S1). One is the effective length, which considers the end correction. The conversion was carried out from a rectangular cross-section to a circular cross-section, maintaining equivalent areas. The parameters approximated and previously assigned are as follows: inner radius of central waveguide r_i, approximated radius of first neck R_{n1} & second neck R_{n2}, and approximated radius of first cavity R_{c1} & second neck R_{c2}. These values result in end-corrected lengths for the first and second necks, denoted as l'_{n1} and l'_{n2}, respectively [S1].

$$l'_{\mathrm{n1}} = l_{\mathrm{n1}} + 0.85 \cdot R_{n1} \cdot \left(1 - 1.25 \cdot \left(\frac{R_{n1}}{r_i}\right)\right) + 0.85 \cdot R_{n1} \cdot \left(1 - 1.25 \cdot \left(\frac{R_{n1}}{R_{c1}}\right)\right), \quad \text{(S1)}$$

$$l'_{\mathrm{n2}} = l_{\mathrm{n2}} + 0.85 \cdot R_{n2} \cdot \left(1 - 1.25 \cdot \left(\frac{R_{n2}}{R_{c1}}\right)\right) + 0.85 \cdot R_{n2} \cdot \left(1 - 1.25 \cdot \left(\frac{R_{n2}}{2}\right)\right), \quad \text{(S2)}$$

The other is effective volume, which accounts for the actual resonating volume. Figure S1 indicates that the second Helmholtz resonator exhibits a significant response specifically at 632 Hz. At this frequency, the resonating volume encompasses both the neck and cavity of the second Helmholtz resonator. The resonating volume of the first Helmholtz resonator comprises not only its own neck and cavity but also the neck of the second Helmholtz resonator. Therefore, in order to accurately calculate the resonance frequency analytically, it would be appropriate to consider these factors in the calculation of the volumes V1 and V2, as outlined in Equation (S3).

$$f_{1,2} = \frac{c_{air}}{2\sqrt{2}\pi}\sqrt{\left(\frac{A_{\mathrm{n1}}}{l'_{\mathrm{n1}}V_1} + \frac{A_{\mathrm{n2}}}{l'_{\mathrm{n2}}V_1} + \frac{A_{\mathrm{n2}}}{l'_{\mathrm{n2}}V_2}\right) \pm \sqrt{\left(\frac{A_{\mathrm{n1}}}{l'_{\mathrm{n1}}V_1} + \frac{A_{\mathrm{n2}}}{l'_{\mathrm{n2}}V_1} + \frac{A_{\mathrm{n2}}}{l'_{\mathrm{n2}}V_2}\right)^2 - 4\frac{A_{\mathrm{n1}}}{l'_{\mathrm{n1}}V_1}\frac{A_{\mathrm{n2}}}{l'_{\mathrm{n2}}V_2}}} \quad \text{(S3)}$$

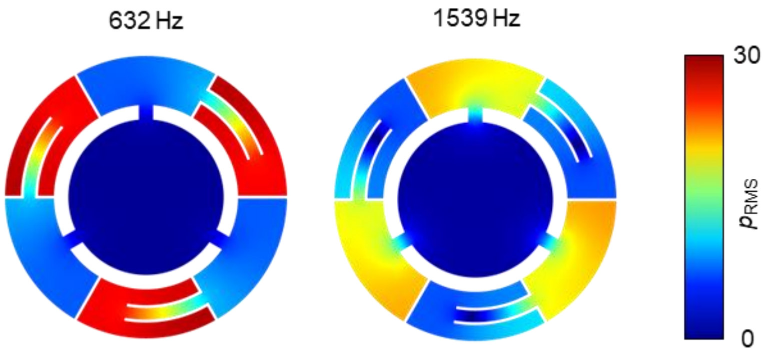

Figure S1. Radial plane view of $D_{in,2}$ layer. The colormap stands for p_{RMS} distribution under the frequency of 632 Hz and 1539 Hz.

S.3 Resonance frequency of the DHRs

The values assigned for $W_{n2,ij}$ in Table S1 lead to the resonance frequency in Table S2, following the Equation S3. This evenly spaced resonance across a broad frequency range results in two effective absorptive bands as discussed in the manuscript.

Table S2. Geometrical parameters of $W_{n2,ij}$ (unit: mm).

$W_{n2,ij}$	i =1	2	3	4	5
j = 1	4	4	4	4	4
2	4.3	4.7	4.6	4.6	4
3	4.7	5.5	5.2	5.2	4

Table S3. Analytically calculated resonance frequency $f_{12,ij}$ (unit: Hz).

$f_{12,ij}$	i =1		2		3		4		5	
j = 1	665	1618	638	1562	608	1499	588	1455		
2	683	1627	675	1585	639	1520	617	1476	568	1415
3	703	1639	709	1613	664	1541	642	1489		

Reference

[S1] Xu, M.B.; Selamet, A.; Kim, H. Dual Helmholtz Resonator. *Appl. Acoust.* **2010**, *71*, 822–829. https://doi.org/10.1016/j.apacoust.2010.04.007.
